# Protein Content and Methyl Donors in Maternal Diet Interact to Influence the Proliferation Rate and Cell Fate of Neural Stem Cells in Rat Hippocampus

**DOI:** 10.3390/nu6104200

**Published:** 2014-10-14

**Authors:** Valérie Amarger, Angèle Lecouillard, Laure Ancellet, Isabelle Grit, Blandine Castellano, Philippe Hulin, Patricia Parnet

**Affiliations:** 1 INRA-University of Nantes, UMR1280, 44093 Nantes, France; E-Mails: angele.lecouillard@oniris-nantes.fr (A.L.); laure.ancellet@elementa-ingredients.com (L.A.); isabelle.grit@univ-nantes.fr (I.G.); blandine.castellano@univ-nantes.fr (B.C.); patricia.parnet@univ-nantes.fr (P.P.); 2 Plateforme MicroPICell, SFR Santé, 44093 Nantes, France; E-Mail: philippe.hulin@nantes.inserm.fr

**Keywords:** neurogenesis, neural stem cells, nutritional programming, methyl donors, epigenetics, hippocampus, insulin, Igf2

## Abstract

Maternal diet during pregnancy and early postnatal life influences the setting up of normal physiological functions in the offspring. Epigenetic mechanisms regulate cell differentiation during embryonic development and may mediate gene/environment interactions. We showed here that high methyl donors associated with normal protein content in maternal diet increased the *in vitro* proliferation rate of neural stem/progenitor cells isolated from rat E19 fetuses. Gene expression on whole hippocampi at weaning confirmed this effect as evidenced by the higher expression of the *Nestin* and *Igf2* genes, suggesting a higher amount of undifferentiated precursor cells. Additionally, protein restriction reduced the expression of the insulin receptor gene, which is essential to the action of IGFII. Inhibition of DNA methylation in neural stem/progenitor cells *in vitro* increased the expression of the astrocyte-specific *Gfap* gene and decreased the expression of the neuron-specific *Dcx* gene, suggesting an impact on cell differentiation. Our data suggest a complex interaction between methyl donors and protein content in maternal diet that influence the expression of major growth factors and their receptors and therefore impact the proliferation and differentiation capacities of neural stem cells, either through external hormone signals or internal genomic regulation.

## 1. Introduction

Pre- and post-natal nutrition are closely associated with brain development, and maternal malnutrition, either global under-nutrition or a lack of specific nutrients, is likely to impact fetal brain development and lead to permanent deficit in learning and behavior [1] or altered control of energy homeostasis [2]. In humans, impaired fetal and postnatal growth, often encountered in preterm infants, represent important risks to develop cognitive difficulties at school age [3,4]. Animal models of under-nutrition or deficiency in specific nutrients were used to demonstrate the profound impact of maternal nutrition during pre-conception, gestation and lactation on the development of the hippocampus and cognitive performance [1], involving the high plasticity of this organ, particularly sensitive to major metabolic sensors, such as insulin or leptin. These hormones are strongly influenced by nutritional status and contribute importantly to neuronal development during fetal and postnatal life, by acting on neurogenesis, axon growth and synaptogenesis [5,6]. We have shown previously in a rat model of nutritional programming that plasma insulin was reduced at weaning in offspring from protein restricted dams, whereas plasma leptin was strongly reduced in response to maternal methyl donor supplementation [7]. We used here the same model to investigate the impact of maternal diet on the development of the hippocampus. Methyl donors and associated cofactors are nutrients involved in the one-carbon metabolic pathway, which plays a major role in neurodevelopment. Maternal zinc, folic acid or choline deficiencies during gestation in rodents were associated with defects in memory for the offspring, together with increased neuronal apoptosis and decreased cell proliferation [8,9,10,11]. Homocysteine was shown to have neurotoxic effects that may be mediated by epigenetic alterations [12]. Dietary intakes are crucial to provide the correct amount of these nutrients, especially during pregnancy and lactation, when the requirements increase, and it was recently shown that these intakes were directly linked to their levels in blood and correlated with DNA methylation at metastable epialleles [13]. These nutrients are crucial to provide methyl groups necessary for a wide range of methylation reactions, including DNA and histone methylation, two epigenetic processes that play a major role in the regulation of developmental genes [14] and particularly in the strict control of cell differentiation during embryo and fetal development of the central nervous system [15,16,17]. Epigenetic mechanisms are also now recognized as key actors in long-term programming effects by providing a link between the early environment and genomic regulation.

In the hippocampus, like in other regions of the mammalian brain, multipotent neural stem cells (NSCs) proliferate through symmetric division and give rise through asymmetric division to neural progenitor cells (NPCs) with a more limited self-renewal potential and a differentiation capacity restricted to either the neuronal or the glial lineage [16]. Neurogenesis can occur in the hippocampus, even in adulthood, where it is involved in learning and memory [18]. A strict control in time and space of the proliferation and differentiation of NSCs is essential to shape the structure and function of the hippocampus. This is closely monitored by a combination of extrinsic neural signals and intrinsic cellular memory that interact through epigenetic mechanisms [16]. While the regulations of NSC differentiation during embryonic and postnatal development have been intensively studied, the impact of nutrients on these processes is still largely unexplored. However, there is growing evidence that environmental cues may modulate the proliferation and/or differentiation capacity of the NSCs by influencing epigenetic memory and, therefore, the cell fate specification. The purpose of this study was to address the influence of maternal diet on these mechanisms.

Considering that the rodent hippocampus develops between Embryonic Day 18 and Postnatal Weeks 2–3 [19], we first studied the effect of fetal nutritional environment on the proliferation and differentiation capacities *in vitro* of NSCs/NPCs collected from the hippocampus at Embryonic Day 19 (E19). Then, we performed an extensive gene expression study on full hippocampi sampled at birth (D0) and weaning (D21) in order to study the impact of maternal diet at two major steps of the development of the organ. The putative role of epigenetic mechanisms was addressed by studying DNA methylation of the *Gfap* gene in D0 and D21 hippocampi [20]. Finally, using specific treatments, we tested the role of DNA methylation on the gene expression pattern of NPCs in culture.

## 2. Experimental Section

### 2.1. Animals and Diets

Animal procedures and maintenance were conducted in accordance with the European Communities Council Directive 2007/526/CE and were approved by the Institut National de la Recherche Agronomique (INRA, Paris, France). The protocol was approved by the local ethics committee for animal experimentation (Comité Régional d’Ethique en Experimentation Animale Pays de Loire) under the license numbers, CEEA.2010.02 and CEEA.2013, and previously described [7]. Two phases of the same protocol were performed using the same experimental design and the same diets. Briefly, virgin female Sprague Dawley rats, 7 weeks old, were fed four different diets for 21 to 28 days before mating and throughout gestation and lactation. The four diets (purchased from Arie Block, Woerden, The Netherlands) consisted of: (1) a control diet (C) containing 20% protein and the minimal required amounts of methyl donors and cofactors (MD); (2) a control MD-supplemented diet (Csup) containing 20% protein and supplemented with an excess of MD (methionine, choline, betaine, folic acid, zinc, B12 vitamin); (3) a protein-restricted diet (R), containing 8% protein and the same amount of MD as the control diet; and (4) a protein-restricted, MD-supplemented (Rsup) diet containing 8% protein and an excess of MD (the detailed composition of the diets is given in Table 1). In the first phase, after delivery, litters were culled to 8 pups per dam (4 males and 4 females), and animals were sacrificed at 21 days of life after overnight fasting (5 to 6 L per group). Hippocampi were quickly dissected, frozen in liquid nitrogen and stored at −80 °C until further RNA and DNA isolation. In the second phase, 3 dams per group were sacrificed at 19 days of gestation (Day 0 being the day after mating), and fetal hippocampi were used for NPCs culture. For the remaining 6 dams per group, gestation was completed, and pups were sacrificed at birth and their hippocampi dissected and snap frozen in liquid nitrogen. For both D0 and D21, only male offspring were used for RNA and DNA isolation.

For reasons of convenience, the cultured cells will be named NPCs in the rest of the article, although, obviously, the cultures contain a mixture of NSCs and NPCs, both of them being present in the hippocampus at the time of sampling and being able to proliferate *in vitro*.

Two additional Sprague Dawley females, one-day pregnant, were purchased from Janvier (Le Genest Saint Isle, France), fed a standard diet (SAFE A03) until D19 of gestation and sacrificed for NPCs culture from fetal hippocampi. These NPCs were used for treatment with a DNA methylation activator or inhibitor, as described below.

**Table 1:** Composition of the diets.

	Control (C)	Control MD-Supplemented (Csup)	Restricted (R)	Restricted MD-Supplemented (Rsup)
Dextrose (%)	10	10	10	10
Sucrose (%)	10	10	10	10
Soybean oil (%)	4.3	4.3	4.3	4.3
Cellulose (%)	5	5	5	5
Corn starch (%)	43.6	37.8	56.6	50.3
Casein (%)	22	22	9	9
Methionine(g/kg)	7.2	12	2.9	12
Choline (g/kg)	1	15	1	15
Betaine (g/kg)	0	15	0	15
Vitamin B12 (mg/kg)	25	1000	25	1000
Folic acid (mg/kg)	2	15	2	15
Zinc (mg/kg)	30	180	30	180
Energy (kcal/kg)	3260.8	3064.6	3261.6	3051.4

### 2.2. Preparation of Rat Hippocampal NPC Cultures

Rat fetuses at D19 of gestation were sacrificed by decapitation, and the brain was removed and kept in ice-cold Hank’s Balanced Salt Solution (HBSS, Life Technologies, Carlsbad, CA, USA) for dissection. The meninges were carefully removed, and the hippocampi were dissected under a binocular microscope. Hippocampi from 2 fetuses were pooled, and 8 fetuses per dams were used (3 dams per diet group). The hippocampi were washed once in 10 mL HBSS and incubated for 15 min at 37 °C in 5 mL HBSS containing 500 µL trypsin/EDTA (0.05%, Life Technologies, Carlsbad, CA, USA). They were washed 6 times with 10 mL HBSS and triturated through a flame-polished Pasteur pipette. The cells were seeded at a density of 2 × 10^5^ cells/mL in TC25 culture flasks containing 10 mL of proliferation medium (Dulbecco’s Modified Eagle Medium (DMEM-F12), 1% antibiotic solution (Pen Strep), 200 mM l-glutamine, 33 mM d-glucose, 2% B27, 20 ng/mL epidermal growth factor (EGF) and 20 ng/mL basic fibroblast growth factor (bFGF); all purchased from Life Technologies, Carlsbad, CA, USA). Cells were allowed to proliferate for 7 days *in vitro* (DIV) under a humidified atmosphere containing 5% CO_2_ at 37 °C. Half of the proliferation medium was replaced every 2–3 days. After proliferation for 7 DIV, the neurospheres were mechanically dissociated by repetitive pipetting. A portion of the cell suspension was seeded at a density of 1 × 10^4^ cells/mL in TC25 culture flasks containing 10 mL of proliferation medium and allowed to proliferate. The flasks were used for neurosphere size quantification. A total of 1188 and 947 neurospheres were measured at 3 and 6 DIV, respectively (about 250 per group at each time point), and they were classified into 50-µm categories. The remaining cells were seeded at a density of 5 × 10^4^ cells/mL in poly-lysine coated 96-well culture plates in differentiation medium (*i.e.*, proliferation medium without EGF and bFGF, 200 µL per well) and allowed to differentiate for 7 DIV, with replacement of half of the medium after 3 days. They were subsequently used for immunocytochemistry experiments.

### 2.3. Immunocytochemistry

Cells attached to poly-lysine-coated 96-well plates were fixed with ice-cold methanol at −20 °C for 15 min, treated with a solution of PBS (phosphate buffer saline) containing 3% BSA (bovine serum albumin) and 0.2% triton, for 30 min and washed with PBS. Cells were incubated with antibodies directed against DCX (1:200 rabbit polyclonal, Abcam, Cambridge, UK), GFAP (1:5000 rabbit polyclonal, Dako, Agilent Technologies, Santa Clara, CA, USA), proliferating cell nuclear antigen (PCNA) (1:200 mouse monoclonal, Santa Cruz Biotechnology, Dallas, TX, USA) or MAP2 (1:100 rabbit polyclonal, Cell Signaling Technology, Danvers, MA, USA) overnight at 4 °C in PBS containing 3% BSA and 0.1% triton. Cells were washed 4 times with PBS and incubated for 90 min at 37 °C with appropriate secondary antibodies: Alexa Fluor goat anti-mouse IgG 488 and Alexa Fluor goat anti-rabbit 488 (all 1:500, Molecular Probes, Life Technologies, Carlsbad, CA, USA). Cells were counterstained with DAPI (Molecular Probes) to visualize the nuclei. Cell counts were performed on a Cellomic ArrayScan VTI HCS Reader (Thermo Fisher Scientific Inc., Waltham, MA, USA) using the Cellomic^®^View and Cell Health Profiling software (Thermo Fisher Scientific Inc., Waltham, MA, USA).

### 2.4. Treatment of Rat Hippocampal NPCs

To determine the effects of SAM (S-adenosyl methionine) and 5-AZA (5-aza-2′-deoxycytidine) treatment on the NPCs *in vitro*, NPCs were prepared from hippocampi of fetuses from two rat females fed a standard diet. For each dam, the hippocampi from 10 fetuses were pooled, and the cells were cultured as described above at a density of 1.5 × 10^5^ cells/mL in proliferation medium for 7 DIV. After passage, the cells were seeded in 5 mL of proliferation medium in 6-well culture plates at a density of 1 × 10^6^ cells/well (9 wells for each initial culture). They were allowed to proliferate for 4 additional days before treatment, with a change of half of the medium after 2 days. After 4 DIV, 6 wells (3 for each initial culture) were treated with SAM 1 mM (Sigma Aldrich, St Louis, MO, USA), 6 wells were treated with 5-AZA 5 µM (Sigma Aldrich, St Louis, MO, USA) and 6 wells did not receive any treatment. The doses of SAM and 5-AZA were determined according to data from the literature demonstrating the absence of toxicity together with the expected biological effects on DNA methylation and DNA methyl-transferase inhibition [21,22,23,24]. After 2 DIV, half of the medium was replaced in each well, and the same treatment was applied for 2 additional DIV. After these 4 days of treatment, the cells were harvested, centrifuged and frozen at −80 °C for subsequent RNA (4 wells per treatment) and DNA isolation (2 wells per treatment).

### 2.5. RT-PCR

Total RNA was extracted from hippocampal neurospheres using a NucleoSpin RNA XS kit (Macherey Nagel, GmbH & Co, Düren, Germany) and from total hippocampi using Qiazol (Qiagen, Venlo, The Netherlands). Two micrograms of RNA were reverse-transcribed into cDNA using Random Primers and Moloney murine leukemia virus (MMLV) reverse transcriptase (Invitrogen, Life Technologies, Carlsbad, CA, USA) in a total volume of 25 µL. Real-time PCR was performed on a 1/40 dilution of cDNA and 2.5 µM of both primers (the primer sequences are given in Table 2) using the iTaq™ Universal SYBR^®^Green Supermix (Bio-Rad, Hercules, CA, USA) following the manufacturer’s instructions, on a CFX Connect^™^ Real Time PCR Detection System (Biorad, Hercules, CA, USA). β-actin (*Actb*) and glyceraldehyde-3-phosphate dehydrogenase (*Gapdh*) were used as reference genes. Relative expression levels normalized to reference genes were expressed as 2^−ΔCt^ × 10^3^ for gene expression in total hippocampi in order to compare the expression level between groups and between development stages and as 2^−ΔΔCt^ for gene expression in cultured cells.

**Table 2:** Primer sequences for quantitative RT-PCR.

Gene	Forward Primer	Reverse Primer
*Actb*	CTATCGGCAATGAGCGGTTCC	GCACTGTGTTGGCATAGAGGTC
*Dcx*	CTTGGATGAGAATGAATGCAGAG	GCTTGTGGGTGTAGAGATAGG
*Dnmt1*	AAACGCAAATGAATCTGCTG	TGTCATCTTCCTGTTCACCT
*Dnmt3b*	CTCATGGAAGATGTGACACCT	AACTCCTTGTCATCCTGATAC
*Gapdh*	CGCCAAGTTCAACGGCACAG	TCCACGACATACTCAGCACCA
*Gfap*	GATCTGGAGAGGAAGGTTGAG	GGGAGTTCTCGAACTTCCTCC
*Igf1*	AAGCCTACAAAGTCAGCTCG	GGTCTTGTTTCCTGCACTTC
*Igf1r*	ATGACACGAGACATCTACGA	TAAGTTCAAACAGCATATCGGG
*Igf2*	AAGTCGATGTTGGTGCTTCTC	GAAGGCCTGCTGAAGTAGAA
*Igf2r*	TGTATCCGTGAACCTGTGTC	AGTTGTCCTCTTCCTGATATTCTG
*Insr*	CATTGTCAGAAAGTTTGCCCA	GGAAGTGATAGTAGGGTGGTG
*Kcnj10*	CTGTGCCAAGATGACATCAG	AACGCTTGTCAGCAATATGC
*Mecp2*	GGACCTATGTATGATGACCC	TCTACTTTAGAGCGAAAGGCT
*Nestin*	ACATACAGGACTCTGCTGGAG	GAAATTCGGCTTCAGCTTGG
*Neun (Rbfox3)*	CTTCCAGGGTCGTGTATCAG	CTCTACCATAACTGTCACTGTAGG

### 2.6. Pyrosequencing

We used pyrosequencing to measure the level of methylation of specific CpG sites in the *Gfap* and *S100β* astrocyte-specific genes. The CpG site (position −1429) in the *Gfap* gene promoter, situated inside a STAT3 binding element (position −1433 to −1425) [20] is known to be demethylated in late gestation in NPCs, allowing the expression of the gene and the differentiation into astrocytes. To the contrary, CpG sites in *Gfap* exon 1 contribute to *Gfap* silencing in neurons by binding the MeCP2 DNA binding protein [25]. The CpG site (position −276 in rat) in the *S100β* rat gene is conserved with the CpG site in position −318 in the mouse gene. This site, when methylated in early gestation, prevents the expression of the gene by binding the MeCP2 DNA binding protein [26].

Genomic DNA was extracted from total hippocampi and from hippocampal neurospheres using proteinase K lysis followed by phenol/chloroform extraction. Two micrograms were submitted to bisulfite conversion using the Methyl Detector bisulfite modification kit (Active Motif Europe, Rixensart, Belgium). Bisulfite-converted DNA was amplified using the Pyromark^®^ PCR kit, and pyrosequencing was performed using the Pyromark^®^ Q24 instrument (Qiagen, Venlo, The Netherlands) following the manufacturer’s instructions. PCR and pyrosequencing primers were designed using the Pyromark^®^ Assay Design software (Qiagen, Venlo, The Netherlands).

The primers sequences for amplification of bisulfite converted DNA were ATGGTTAGGGGAGGGTATATAGTTTGAT and CCTTAATACAATACAAACTCCCAATCTA for the *Gfap* promoter; GGAGTTAGTAGAAGTAGGGTAAGAT and ATAAAACCATAACCCCTAACCA for *Gfap* exon 1; and AGGTTTTTTTGGAAGTTAGTTATAGTTA and AACAACCACAATTAAATCTAATCT for the *S100β* promoter. The primers used for the pyrosequencing reaction were TGTATTTTAGGTTTTTTTTAATGTT, AAGTAGGGTAAGATGGA and GGTTGAGTATTTGTTGTTTGAATTA for the *Gfap* promoter, *Gfap* exon 1 and the *S100β* promoter, respectively.

### 2.7. Statistical Analysis

Data were analyzed using GraphPad Prism^®^ 5 (GraphPad software Inc., La jolla, CA, USA). The distribution of the neurosphere circumference was tested using the chi-square test. For immunocytochemistry, gene expression and DNA methylation data, comparison between the four or three different groups was performed using the Kruskal–Wallis test followed by Dunn’s multiple comparison tests, which adjusts for multiple testing. The individual effect of protein restriction and MD supplementation and the interaction between these two parameters were tested using two-way ANOVA. *p* < 0.05 was considered as significant.

## 3. Results

### 3.1. Methyl Donor Supplementation Associated with Normal Protein Content Increased NPC Proliferation Rate in Vitro and Promoted Neuronal Differentiation

**Figure 1:**
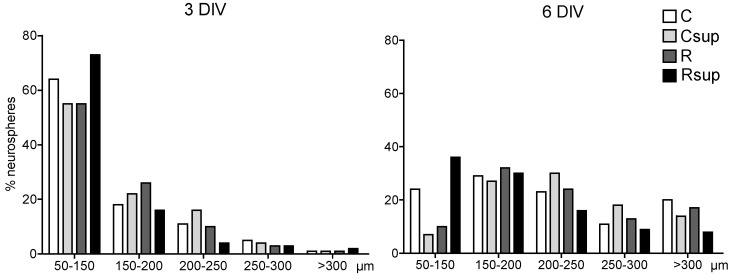
Neurosphere circumference after three and six days *in vitro* (DIV). The data represent the % of neurospheres in each size category in relation to the total number of neurospheres that was measured (around 250 per group at each time point, originating from four different cultures from each female, three females per group). C, control diet; Csup, control MD-supplemented diet; R, protein-restricted diet; Rsup, protein-restricted, MD-supplemented diet.

The size of the neurospheres ranged from 50 to 400 µm after three DIV and from 100 to 450 µm after six DIV (Figure 1). The distribution of the circumferences between the four groups was significantly different both at three DIV and six DIV (chi-square *p* < 0.0001). The proportion of large neurospheres (>200 µm) at six DIV was higher in the Csup group compared with the C and Rsup groups, but the distribution was rather similar between the Csup and R groups. At both times, the Rsup group had the highest proportion of neurospheres <200 µm (about 90% at three DIV and 65% at six DIV), whereas the Csup group had the lowest (77% at three DIV and 34% at six DIV).

**Figure 2:**
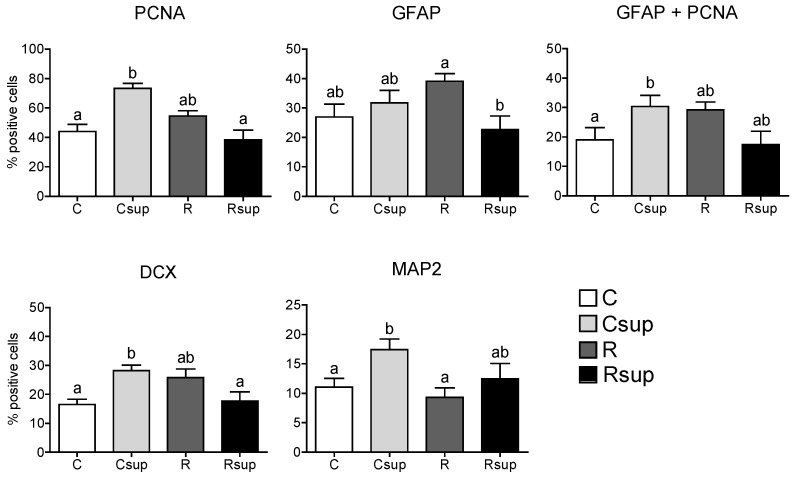
Proliferation and differentiation markers in NPC cells cultured *in vitro* after seven DIV on differentiation medium. Data are presented as % of positive cells (mean ± SEM, *n* = 12 different cultures per group, which correspond to four pools of two embryos per female, three females per group). ^a,b^ Different superscripts indicate statistically significant difference between groups (Kruskal–Wallis and Dunn’s *post hoc* tests).

After seven DIV on differentiation medium, about 75% of the cells from the Csup group expressed the PCNA marker, significantly (*p* < 0.05) more than the other groups (Figure 2). The proportion of cells expressing GFAP was similar between the C, Csup and R groups and lower in the Rsup group compared with R (*p* < 0.01, Dunn’s test). However, in the Csup group, all cells expressing GFAP co-expressed PCNA (Figure 2), suggesting that they correspond to glial progenitors, whereas, in the other three groups, about ¼ of the GFAP positive cells did not co-express PCNA and, therefore, were likely to be differentiated astrocytes. MAP2 was expressed in a higher number of cells in the Csup group compared with the C and R groups, suggesting a higher number of mature neurons. Altogether, these data suggested a higher number of proliferating cells and a preferential differentiation in neurons in the Csup group, whereas, cells from the C, R and Rsup groups seemed to enter the differentiation process towards the astrocyte lineage earlier.

### 3.2. Protein Content and Methyl Donors in Maternal Diet Interacted to Influence Gene Expression in Whole Hippocampus at Weaning

**Figure 3:**
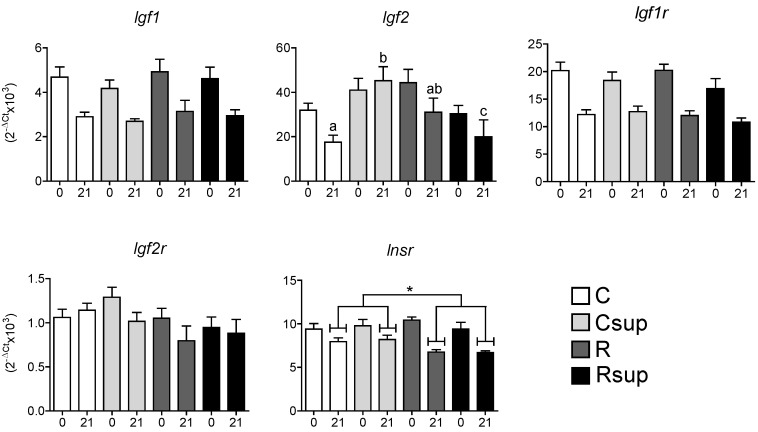
Relative gene expression in hippocampus at D0 (0) and D21 (21). Data are presented as the mean ± SEM (*n* = 12 at D0 and *n* = 8 at D21). ^a,b^ Different superscripts indicate a statistically significant difference between groups at the same age; the absence of a superscript indicates that there is no statistically significant difference between groups at the same age (Kruskal–Wallis and Dunn’s *post hoc* tests), * *p* < 0.05 (two-way ANOVA).

In order to assess whether the observed differences in the proliferation and differentiation capacities of NPCs *in vitro* had influenced the development of the hippocampus *in vivo,* the expression of a set of genes encoding growth factors involved in proliferation and genes specifically expressed in the different cell lineages was measured in whole hippocampi sampled at birth and at weaning, two different stages of maturation of this organ [27]. The expression of the *Igf1* and *Igf1r* genes decreased almost two-fold between D0 and D21, but there was no difference between the four groups at any time (Figure 3). The *Igf2* gene was strongly expressed at D0 compared with *Igf1* and its expression decreased between D0 and D21. This was similar between the four groups at D0 and between the C and R groups at D21. Surprisingly, *Igf2* expression was increased about three-fold in the Csup group compared with the C group, whereas it was reduced in the Rsup group compared to the R group. Indeed, the data analysis using two-way ANOVA revealed a significant interaction between the MD supplementation and protein content in maternal diet (*p* = 0.003), although both factors have no independent effect on *Igf2* expression.

The *Igf2r* gene was expressed at rather low levels compared with *Igf1r* at both stages, and there was no difference, either between groups or between stages. The expression of the *Insr* gene slightly decreased between D0 and D21 and was significantly reduced in the protein-restricted groups (*p* < 0.01, two-way ANOVA) at D21, but not influenced by methyl donor supplementation. The same tendency was observed for the *Igf2r* gene, but did not reach significance (*p* = 0.07).

**Figure 4:**
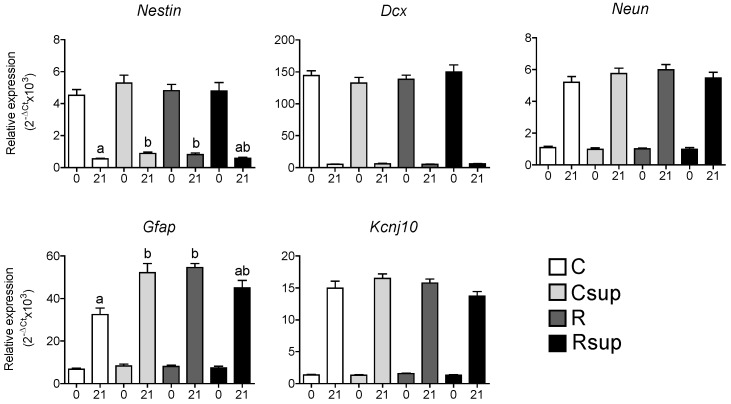
Relative gene expression in hippocampus at D0 (0) and D21 (21). Data are presented as the mean ± SEM (*n* = 12 at D0 and *n* = 8 at D21). ^a,b^ Different superscripts indicate a statistically significant difference between groups at the same age; the absence of a superscript indicates that there is no statistically significant difference between groups (Kruskal–Wallis and Dunn’s *post hoc* tests).

The evolution of the expression level of lineage-specific genes between D0 and D21 was indicative of the maturation of the hippocampus (Figure 4). We observed a strong reduction in the expression of *Nestin* and *Dcx*, which are specific to undifferentiated cells and immature neurons, respectively. In addition, an increase in the expression of *Neun* and *Kcnj10*, specifically expressed in mature neurons and astrocytes, respectively, was observed. The *Gfap* gene, expressed both in glial progenitors and mature astrocytes, showed a strong increase of expression between D0 and D21. At birth, the expression of all of these genes was not different between groups. At weaning, *Nestin* and *Gfap* were overexpress about 1.5-fold in the R group compared with the control group, indicating an effect of maternal protein restriction. Surprisingly, the MD supplementation was also associated with an increase in the expression of these two genes when combined with normal protein content (Csup group), whereas it had no effect when combined with the reduced protein content (Rsup group). The interaction between protein content and MD supplementation was considered highly significant for both genes (*p* = 0.0003 for *Nestin*, *p* = 0.0001 for *Gfap*, two-way ANOVA).

**Figure 5:**
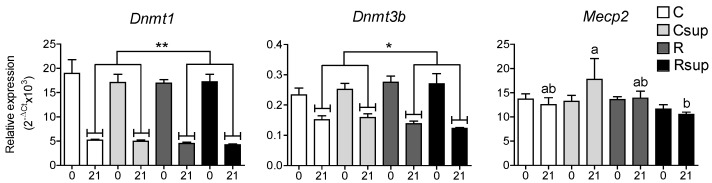
Relative gene expression in hippocampus at D0 (0) and D21 (21). Data are presented as the mean ± SEM (*n* = 12 at D0 and *n* = 8 at D21). ^a,b^ Different superscripts indicate a statistically significant difference between groups at the same age; the absence of a superscript indicates that there is no statistically significant difference between groups (Kruskal–Wallis and Dunn’s *post hoc* tests) * *p* < 0.05, ** *p* < 0.01 (two-way ANOVA).

Since we expected the supplementation in MD to influence DNA methylation levels in offspring, we measured the expression level of genes encoding the DNA methyltransferases, DNMT1 and DNMT3B, as well as the DNA binding protein, MeCP2, which is involved in the silencing of astrocyte-specific genes in neurons [28]. The *Dnmt1* gene was highly expressed at birth compared with the *Dnmt3b* gene, and the expression of both of them decreased between D0 and D21. For both genes, there was no difference between groups at D0 and a slight, but significant under-expression for the protein-restricted groups (R and Rsup) at D21 (*p* = 0.008 for *Dnmt1* and *p* = 0.01 for *Dnmt3b*, two-way ANOVA) (Figure 5). The expression of the *Mecp2* gene did not vary between D0 and D21, except for the Csup group, which showed an overexpression at D21, compared with the Rsup group.

### 3.3. DNA Methylation of the Gfap Gene in Whole Hippocampus Changed between Birth and Weaning, but Was Barely Affected by Maternal Diet

**Figure 6:**
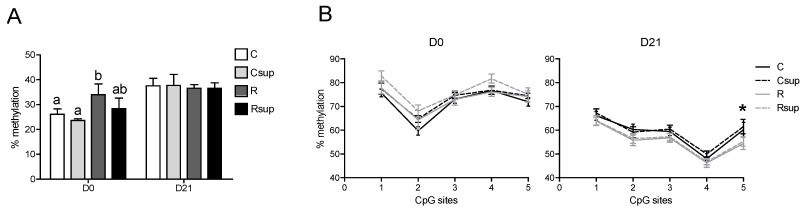
DNA methylation analysis of the *Gfap* gene promoter and exon 1 in whole hippocampi sampled at birth (D0) and weaning (D21). (**A**) The methylation level of the CpG site at position –1429 in the *Gfap* promoter. (**B**) The methylation level of five CpG sites at positions 8, 14, 29, 32 and 49 in *Gfap* exon 1. ^a,b^ Different superscripts indicate a statistically significant difference between groups at the same age; the absence of a superscript indicates that there is no statistically significant difference between groups (*n* = 6, Kruskal–Wallis and Dunn’s *post hoc* tests); * *p* < 0.05 (two-way ANOVA).

The CpG site in the *Gfap* promoter showed a methylation level varying from 25% to 35% in hippocampi sampled at birth and around 40% at weaning (Figure 6A). At birth, the R group harbored a significantly higher methylation level compared with the C group, but the MD supplementation did not show any effect, either in the Csup or in the Rsup groups compared to C and R, respectively. At weaning, there was no longer any difference between the four groups. The methylation level of the five CpG sites in *Gfap* exon 1 was heterogeneous and decreased between D0 and D21, varying from 60% to 80% at birth and from 45% to 70% at weaning (Figure 6B). Moreover, the methylation profile of this region was different between birth and weaning. For instance, the CpG No. 4 was about 70% to 80% methylated at birth and only 40% to 50% at weaning (Figure 6B). The methylation level of the five CpG sites at D21 was lower in the R and Rsup groups compared with the C and Csup groups, but it was significant for the CpG site No. 5 only (*p* = 0.03, two-way ANOVA).

### 3.4. Influencing DNA Methylation of NPCs in Vitro Affects the Expression of Lineage-Specific Genes

**Figure 7:**
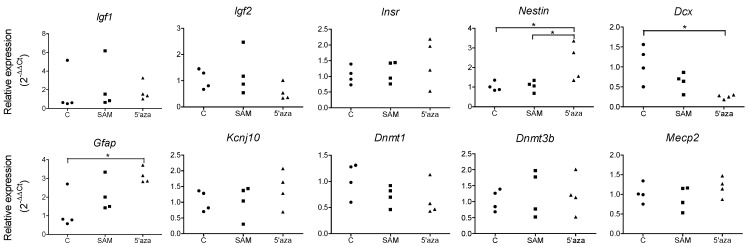
Relative gene expressions in cultured NPCs. Data are presented as scatter plots, each point representing the cells from one well (six-well culture plates). For each treatment group, two wells contained NPCs cultured from the pool of 10 embryos from one female rat and two wells from another female. (* *p* < 0.05, Kruskal–Wallis and Dunn *post hoc* tests).

We next sought to establish whether influencing DNA methylation during the proliferation of neural stem cells *in vitro* could affect gene expression and influence their differentiation potential. For that purpose, we submitted NPCs in cell culture to a treatment with SAM, which is a methyl group provider, or 5-AZA, an inhibitor of the DNA methyl transferases. We observed an increase in the expression of the *Nestin* and *Gfap* genes in the 5-AZA-treated cells, whereas the *Dcx* gene was underexpressed (Figure 7). There was no significant difference in the expression of the *Igf1*, *Igf2*, *Insr*, *MecP2*, *Kcjn10* and *Dnmt3b* genes. As expected, the expression of the *Dnmt1* gene was reduced, but only in three out of four wells for the 5-AZA group (Figure 7).

**Figure 8:**
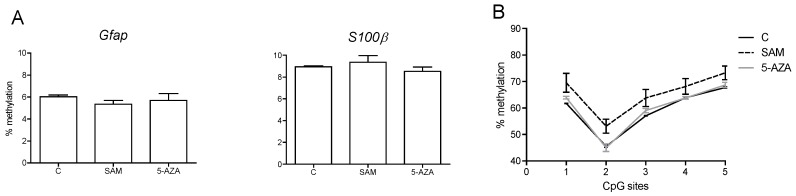
DNA methylation analysis of the *Gfap* and *S100β* genes in cultured NPCs treated with SAM and 5-AZA. (**A**) The methylation level of the CpG sites at position –429 in the *Gfap* promoter and –276 in the *S100β* promoter. (**B**) The methylation level of five CpG sites at positions 8, 14, 29, 32 and 49 in *Gfap* exon 1. (*n* = 2 different well cultures per treatment).

The two CpG sites situated in the *Gfap* and *S100β* gene promoters were hypomethylated (around 5% and 9% for *Gfap* and *S100β*, respectively) (Figure 8), and there was no difference between the treatments; however, because of the low number of cultures (*n* = 2 per treatment), we did not perform any statistical analysis. The methylation profile of the *Gfap* exon 1 on cultured cells was identical to the one observed in the hippocampus at birth, although the overall methylation level was lower (Figure 8). The cells treated with SAM had a slightly higher level of methylation for the five CpGs, whereas there was no difference between the control and 5-AZA-treated cells.

## 4. Discussion

This study was designed to test the hypothesis that maternal nutrition during the preconception, gestation and lactation periods may influence the development of the hippocampus in the offspring. We combined an *in vitro* approach on fetal hippocampus neural stem/progenitor cells obtained at the end of gestation with a gene expression analysis on whole hippocampi at birth and weaning. This strategy addressed the effect of the pre- and post-natal nutritional environment on the memory of undifferentiated cells when cultured *in vitro* and on the development of the whole hippocampus *in vivo*.

NPCs sampled on E19 hippocampi are able to proliferate and differentiate *in vitro* keeping in memory some reminiscent characteristics of their previous environment. That was indeed demonstrated here, since NPCs from the group supplemented in methyl donors (Csup) had a higher proliferation rate *in vitro* and differentiated preferentially in neurons, whereas NPCs from the protein-restricted groups did not differ from the control group, clearly suggesting that only the supplementation in MD had an impact on the memory of the cells. Yet, fetal protein restriction is known to induce alterations in brain development [29,30], and a 50% caloric restriction during fetal life was shown to reduce the proliferation of adult generated hippocampal cells [31]. The protein restriction may therefore impact the organ development *in vivo* via external cues rather than the intrinsic program of the cells or, alternatively, could act by conditioning the cell response to external cues that are not present when the cells are cultured *in vitro*.

Therefore, we performed a gene expression study at D0 and D21 in order to reveal how the developing hippocampus was affected by its fetal and postnatal environment *in vivo*. At birth, the hippocampus is immature, and postnatal life is characterized by intensive migration, rearrangement and maturation of neurons, dendritogenesis and synaptogenesis [32]. Therefore, as expected, genes expressed in undifferentiated or immature cells, such as *Nestin* or *Dcx*, were underexpressed at D21 compared with D0, whereas genes expressed in mature neurons (*Neun*) or astrocytes (*Kcnj10*) were overexpressed at D21.

We observed that the maternal protein restriction was associated with a decreased expression of the *Insr* gene and that the MD supplementation had no effect, whereas the expression of the *Igf2* gene was not affected by the protein restriction alone, but rather by an interaction between protein content and MD supplementation. Previous work had shown that the expression of *Igf2* in hippocampus was stimulated by a prenatal supplementation in choline, which was one of the MDs of our diet [33]. Insulin-like growth factors (IGFI and IGFII) and insulin are major regulators of brain growth, differentiation and maintenance [27]. Insulin was shown to stimulate NPCs proliferation *in vitro* [34,35], and IGFII selectively controls the proliferation of hippocampal dentate gyrus NSCs *in vitro* and *in vivo* [36].

It is now established that neonatal protein restriction impairs beta-cell development and insulin secretion [37], and we observed that offspring from the R and Rsup groups had low levels of plasmatic insulin at weaning [7], which correlates with the low expression of the *Insr* gene and suggests that the amount of receptor in the brain somehow adapted to the lower level of circulating insulin. Carbohydrate content also differs between the diets containing 20% *vs.* 8% proteins in order to compensate for the caloric loss. However, it was demonstrated that the maternal glycemia and insulinemia were not disturbed in this type of model [38,39], sustaining the fact that the observed effects on the offspring are not a consequence of an impaired maternal insulin metabolism during gestation, but rather the consequence of the shortage in amino acid supply.

However, the insulin receptor also binds IGFII, and it was demonstrated that this interaction was necessary to maintain a pool of NSCs in the brain, whereas the IGF1R was more involved in NPC proliferation [35]. In our model, the Csup group was the only one to combine normal plasmatic insulin levels [7] and higher *Igf2* and *Insr* gene expression in hippocampus, whereas the control group had a similar insulin level, but lower *Igf2* expression. Additionally, the R and Rsup groups had low insulin and low expression of *Insr* and *Igf2* (especially Rsup). This complex interaction between these different growth factors may explain the complexity of the interaction between protein and methyl donor content in the nutritional environment.

The increased expression level of the *Igf2* gene in the Csup group at weaning, associated with an increased expression of the *Nestin* gene, is also indicative of the presence of a larger pool of undifferentiated cells in the Csup group, which is consistent with the enhanced proliferation capacity of the NPCs.

Altogether, these observations indicate a combined effect of the MD supplementation and the reduced proteins via specific ways: the MD supplementation would increase the proliferation capacity of the neural stem/progenitor cells, possibly by stimulating the expression of *Igf2*, but the protein restriction would reduce the availability of insulin and the insulin receptor, which are essential to promote proliferation and neural differentiation. That would explain why the MD supplementation had no effect on proliferation capacity when combined with the protein restriction.

The implication of epigenetic mechanisms in the differentiation capacity of neural stem cells is widely admitted, and DNA methylation plays a major role in the control of gene expression that determines the timing of differentiation, as well as cell fate [26,40,41]. Maternal protein restriction was shown to slightly reduce the expression of the *Dnmt1* and *Dnmt3b* genes at weaning, as well as the methylation level of the *Gfap* exon 1, but we did not show any evidence of an impact of MD supplementation.

However, when DNA methylation was monitored *in vitro* by treating the NPCs with SAM or 5-AZA, we did observe several effects that suggest that DNA methylation influences the differentiation potential of the NPCs. For instance, the treatment with SAM was associated with a slight increase in the methylation level of the *Gfap* exon 1. As it is known that the methylation of this region allows the binding of the MecP2 protein in neurons in order to inhibit *Gfap* expression, this result may reflect a higher propensity of the NPCs treated with SAM to differentiate into neurons. On the other hand, we showed here that treatment of NPCs with DNA methyltransferase inhibitor reduced *Dcx* expression and enhanced *Gfap* expression, demonstrating a preferential differentiation towards the astrocyte lineage. These observations are in agreement with the preferential *in vitro* differentiation of the NPCs from the Csup group in neurons; this was emphasized by published data showing that folic acid induced NSCs differentiation into neurons *in vitro* [42]. We did not show evidence of a higher amount of neurons in the hippocampi *in vivo*, but the age of 21 days might be too early. Since neurogenesis occurs in hippocampus even in adulthood [18], we can hypothesize that the programming effect of early nutrition will affect adult rather than pre-weaning neurogenesis.

## 5. Conclusions

Our results suggest that maternal nutrition, such as protein restriction and methyl donor supplementation, may impact hippocampus development in offspring by: (1) influencing gene expression at the cellular level and, therefore, affecting the proliferation capacity of neural stem/progenitor cells; and (2) regulating the synthesis of major metabolic sensors, such as insulin and IGFII.

Importantly, our results clearly indicate that proteins and nutrients involved in the folate metabolic pathway interact and that their effects are strongly dependent on each other’s availability. Further data are needed to establish the implication of an epigenetic mechanism in the observed effects, and morphological, as well as functional analyses are required to confirm these data.
